# Temperature and interlayer coupling induced thermal transport across graphene/2D-SiC van der Waals heterostructure

**DOI:** 10.1038/s41598-021-04740-4

**Published:** 2022-01-14

**Authors:** Md. Sherajul Islam, Imon Mia, A. S. M. Jannatul Islam, Catherine Stampfl, Jeongwon Park

**Affiliations:** 1 Department of Electrical and Electronic Engineering, Khulna University of Engineering andTechnology, Khulna, 9203 Bangladesh; 2 School of Physics, The University of Sydney, Sydney, NSW 2006 Australia; 3 Department of Electrical and Biomedical Engineering, University of Nevada, Reno, NV 89557 USA; 4 School of Electrical Engineering and Computer Science, University of Ottawa, Ottawa, ON K1N 6N5 Canada

**Keywords:** Engineering, Materials science, Nanoscience and technology, Physics

## Abstract

Graphene based two-dimensional (2D) van der Waals (vdW) materials have attracted enormous attention because of their extraordinary physical properties. In this study, we explore the temperature and interlayer coupling induced thermal transport across the graphene/2D-SiC vdW interface using non-equilibrium molecular dynamics and transient pump probe methods. We find that the in-plane thermal conductivity *κ* deviates slightly from the 1/*T* law at high temperatures. A tunable *κ* is found with the variation of the interlayer coupling strength χ*.* The interlayer thermal resistance *R* across graphene/2D-SiC interface reaches 2.71 $\times$ 10^–7^ ${\text{Km}}^{2} /{\text{W}}$ at room temperature and χ = 1, and it reduces steadily with the elevation of system temperature and χ, demonstrating around 41% and 56% reduction with increasing temperature to 700 K and a χ of 25, respectively. We also elucidate the heat transport mechanism by estimating the in-plane and out-of-plane phonon modes. Higher phonon propagation possibility and Umklapp scattering across the interface at high temperatures and increased χ lead to the significant reduction of *R*. This work unveils the mechanism of heat transfer and interface thermal conductance engineering across the graphene/2D-SiC vdW heterostructure.

## Introduction

The performance of nano-electronics, optoelectronics, and phononic devices relies strongly on the efficiencies of heat energy dissipation^1–3^. The effectiveness of heat energy dissipation is firmly based on phonon transportation ability because phonons are the key thermal energy carriers in semiconducting and insulating materials^4,5^. Overpopulated phonons can be caused by the high electrical flow of current, which in effect dramatically enhances electron–phonon scattering as well as phonon–phonon scattering. These processes play into the joule heating effects of regional heat localization and develops local thermal hot spots^6,7^. Failure to effectively dissipate such a robust amount of generated heat will result in reduced performance, types of defects, and even material disruption. For effective development and design of nano-electronic and optoelectronic devices, it is therefore very important to know the device heat transport characteristics. Of late, two-dimensional (2D) materials have acquired enormous interest with regard to solving the problem of heat-energy dissipation and providing an efficient approach to handle the thermal transport phenomenon in nanoscale devices^8–17^. 2D materials offer appealing electronic, mechanical, and phononic properties, which are highly demanded for nano-electronics, optoelectronics, and phononic devices^18–23^.

On the other hand, advanced structures of 2D materials where two or more atomic sheets are held together by a van der Waals forces, and thus form hybrid structures known as van der Waals heterostructures (vdWHs), can retain their all stacked monolayer properties^24–33^. In this regard, numerous vdWHs have been proposed using graphene and related 2D materials with superior electronic properties, exceptional thermal characteristics, unique mechanical properties as well as other physical properties. In particular, the graphene/2D-SiC vdWH has recently been explored with exotic thermal behavior and unique electronic properties being reported^2,28^. In our earlier study, we also reported that graphene/2D-SiC^2^ shows exceptional thermal transport across the interface to mitigate the heat dissipation problem in next generation nanoelectronic devices. However, thermal transport at the nanoscale shows diverse temperature dependent behaviors^34–36^. At high temperatures, the thermal twitching characteristics increase considerably and therefore phonon anharmonicity increases significantly, leading to a decrease in thermal conductivity *κ*. Usually, the *κ* obeys the *1/T* rule in single atomic crystals such as graphene, phosphorene, and stanene at high temperatures^11,37–39^. However, some binary atomic structures exhibit a peculiar, slowly diminishing characteristic of *κ* with increasing temperature owing to the overwhelming impact of high-frequency optical phonons^35,36^. It is thus crucial to know what types of temperature dependent behavior could be found from this promising graphene/2D-SiC vdWH. In addition, the compact structure of thermal interface materials in vdWHs may change the contact pressure variations, which can significantly hinder the thermal transport performance^40–43^. Along with the temperature effects, a detailed insight into contact pressure effects is also required to efficiently lower the thermal resistance across graphene/2D-SiC interface.

Here, we explore the temperature and interface contact pressure effects on thermal transport across the graphene/2D-SiC interface employing reverse non-equilibrium molecular dynamics (RNEMD) simulations and the transient pump–probe (TPP) method. The thermal resistance across the graphene/2D-SiC interface and in-plane thermal conductivity have been evaluated by varying the temperature from 100 to 700 K and the coupling strength from 0.1 to 25. When estimating thermal conductivity at lower temperatures, classical MD simulations neglect quantum effects. The thermal transport at the interface incorporating the quantum correction effect is also investigated. Furthermore, the key mechanisms of heat transfer in the in-plane and out-of-plane directions of the heterobilayer are explored estimating various phonon modes.

## Computational details

The graphene/2D-SiC heterostructure was built using a supercell consisting of a lateral periodicity of 2 × 2 for graphene and 3 × 3 for 2D-SiC, respectively. To maintain commensurability, the graphene/2D-SiC lattice parameter was set to $a_{{{\text{C}}/{\text{SiC}}}} = 3a_{{{\text{SiC}}}} = 9.285 {\text{\AA}}$, imposing a 1% tensile strain on graphene. To attain a large structural system, the supercell structure was repeated in the x and y axes. The interlayer interaction between two layers was described using the Lennard Jones (LJ) potential according to

1 $$ V\left( r \right) = 4{\upchi }\varepsilon \left[ {\left( {\frac{\sigma }{r}} \right)^{12} - \left( {\frac{\sigma }{r}} \right)^{6} } \right]; r < r_{c} , $$

where $r$, $\varepsilon$, *r*_*c*_, and $\sigma$ denote the interatomic distance, energy, cut off distance and length parameters, respectively. The interface interaction parameter is indicated by *χ*, and it may be changed to control the interface strength. The parameters $\varepsilon_{C - C}$ = 4.560 meV, $\varepsilon_{Si - C}$ = 8.909 meV, $\sigma_{C - C}$ = 3.431 Å and $\sigma_{Si - C}$ = 4.067 Å were chosen from Ref^44^.The interlayer coupling ${\upchi }$ and cutoff distance were set at 1 and 10 Å, respectively. Initially, the interlayer separation between graphene and 2D-SiC layer was selected to be 3.4 Å. The spacing, however, is automatically changed after the structure is relaxed. All the directions were subjected to periodic boundary conditions (i.e., *x*, *y*, *z*) to eradicate the edge effect. A 10 nm thick vacuum distance in the *z*-direction was also used to remove atomic contact with the system image. The dimension of the structure considered here was 100 × 10.8 ${\text{nm}}^{2}$ (length × width). All the calculations were carried out in the Large Scale Atomic/Molecular Massively Parallel Simulator (LAMMPS) package^45^. Optimized Tersoff potentials formulated by Lindsay et al.^46^ and Erhart et al.^47^ were employed to explain C–C and Si–C interactions in the graphene and SiC layers, respectively. These potentials were previously utilized to accurately measure the thermal transport behavior of graphene and 2D-SiC.

To quantify the in-plane thermal transport, the RNEMD simulations^48^ were carried out in two phases: the relaxation of the system and the thermal foisting steps. The structure was first brought to an equilibrated position at the desired temperature during the relaxation process. After minimizing the energy with the Conjugated Gradient Algorithm, an isothermal-isobaric (NPT) ensemble simulation was performed for 10^5^ time steps to tune the system to the desired pressure and temperature, where each time step ∆t was 0.5 fs. The velocity-verlet integration method was employed to reach the required temperature. The initial temperature was then raised to the target temperature using 50 ps of microcanonical (NVE) ensemble simulation with a time step of 10 ps. As the temperature was shifted to a new value, the temperature and pressure of the system were further stabilized by applying an additional NPT ensemble simulation for 100 ps. The structure was then further subjected to an additional NVE ensemble simulation for 400 ps to check whether the system was relaxed or not and to stabilize the conserved energy. If the temperature of the system does not change with time, indicating that the system is equilibrated at that particular temperature. The quantitative atomic velocity distribution in the system was further estimated and compared to the Maxwell–Boltzmann predictions to confirm that the structure becomes stable. If the structure becomes stable, the velocities of the atoms in the heterostructure follow the Maxwell–Boltzmann distribution as follows:

2 $$ P_{M } = 4\pi v^{2} \left( {\frac{m}{{2\pi K_{B} T}}} \right)^{\frac{3}{2}} e^{{ - \frac{{mv^{2} }}{{2K_{B} T}}}} $$

here,$P_{M}$,*ν*, $K_{B}$, and *m* represent the probability*,* velocity, Boltzmann constant, and mass of the atom. We also monitor the process temperature over time to ensure that the temperature stays stable. After the system has been relaxed, the NEMD simulation was done to determine the in-plane thermal conductivity of the graphene/2D-SiC vdWH using Fourier's law as follows:

3 $$ k_{x} = - \frac{{J_{x} }}{\nabla T} $$

where $$ \nabla T$$ is the temperature gradient, and $$ J_{x}$$ signifies the *x*-direction heat flux. A hot and cold chunk with a width of 20 Å was introduced in the system positioning at *L*/4 and 3*L*/4 locations, respectively, where *L* represents the length. The heat flux passing through the system can be computed as $$J_{x} = \frac{{\Delta {\upvarepsilon }}}{2AMt}$$, where *Δε* is the energy difference of the thermostat at every *M (*heat exchange frequency) time steps*,* and *A* = *wh*, is the cross-sectional area. The value of M was set to 1000 (500 fs)^7,34^. *t* denotes the time step of the MD simulations. Following the imposition of heat energy, the time-averaged temperature distribution was calculated to perform NVE ensemble simulation for an additional $$24 \times 10^{6}$$ time steps (i.e., 1200 ps) from which $$\Delta T$$ was computed.

Finally, the thermal transport across interface was investigated by calculating the interfacial thermal resistance *R*. We employed TPP to probe the *R* at the heterobilayer interface^49^. The schematic of the TPP technique is portrayed in Fig. S1 (supplementary information). The interface heat transport for several vdWHs has been evaluated by this technique in past studies^40–43^. The system was relaxed at a specific temperature to calculate the *R* across the graphene/2D-SiC interface using the same steps as the *κ* measurement portion. Following the system's relaxation, the graphene layer was subjected to a 50 fs ultrafast heat pulse. As a result, the graphene layer's temperature rises, while the 2D-SiC layer's temperature stays unchanged. Once the heat impulse is withdrawn, the graphene temperature will gradually drop, while the temperature of the SiC sheet would gradually rise. The temperature evolution of graphene (*T*_G_) and 2D-SiC (*T*_2D-SiC_) was tracked throughout the entire thermal equilibrium procedure. At each step, the total energy of graphene (*E*_*t*_) was also collected in order to calculate the *R* according to^2^

4 $$ \frac{{\partial E_{t} }}{\partial t} = \frac{{A_{R } \cdot \left( {T_{G} - T_{2D - SiC} } \right)}}{R} $$

where *A*_R_ is the graphene/2D-SiC contact area. To reduce data noise, the data points acquired at each time step were averaged after every 100 time steps.

## Results and discussion

**Figure 1:**
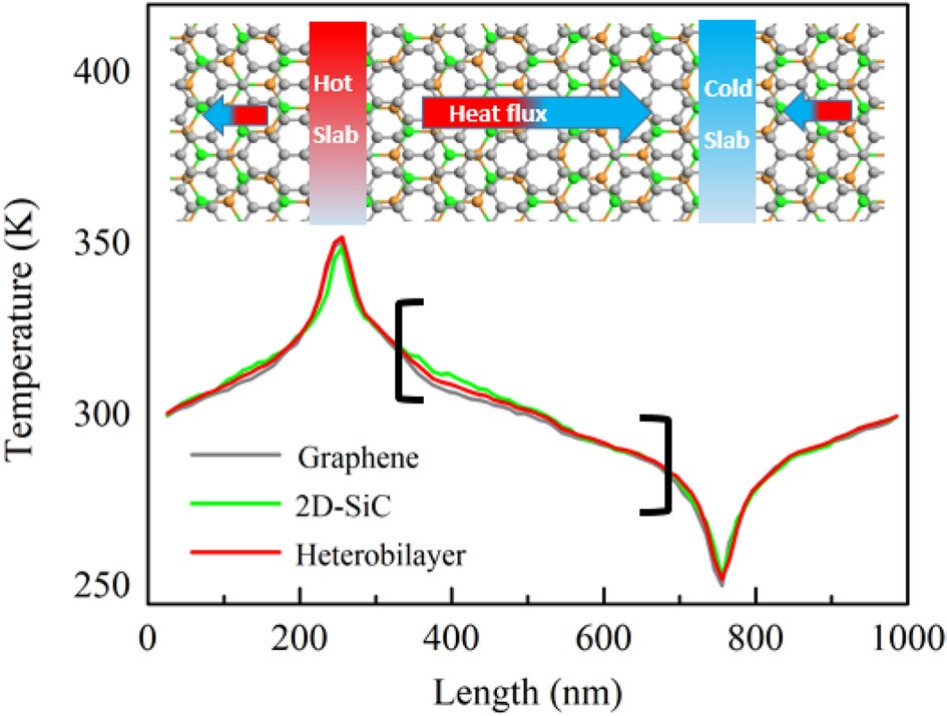
Atomic configuration and NEMD application procedure for the graphene/2D-SiC hetero-bilayer. Periodic boundary conditions are applied in the lateral *x* and *y* directions. Free boundary condition is used in the *z* direction. Temperature distributions of graphene, 2D-SiC and bilayer at steady state.

Observing the time dependent system temperature and the atomic velocities, the stability of the developed vdWHs was confirmed at steady-state conditions. As revealed in Fig. S2a (supplementary information), the temperature does not change with time during the relaxation state, especially in the NVE ensemble state, indicating that the structure is in thermal equilibrium. Figure S2b (supplementary information) shows a comparison of the graphene and 2D-SiC atomic velocity distributions with the Maxwell theoretical prediction. The MD simulation results agree with the Maxwell distribution very precisely, which further justifies that the structure is in thermal equilibrium or steady-state condition. The projected temperature distribution profile at steady-state along the x-direction of the heterobilayer is presented in Fig. 1. Here, *T* + *∆T* and *T-∆T* are used to control the temperature of the hot and cold chunks, respectively, where *∆T* is around 50 K. A rapid temperature fall can be seen near the heating and cooling areas. Similar results have been obtained in NEMD simulations for a variety of systems^6,34^. There are rapid energy transfers between potential and kinetic energies near the hot and cold points. ∇*T* is calculated from the average temperature of the regions indicated by the black brackets as displayed in Fig. 1. In contrast to heterobilayer, graphene and 2D-SiC temperature distributions are also shown, demonstrating the distribution of heterostructure stays within the graphene and 2D-SiC layers. Graphene, 2D-SiC, and the heterobilayer ∇*T’s* are calculated to be 0.336, 0.426, and 0.378 K/nm, respectively.

**Figure 2:**
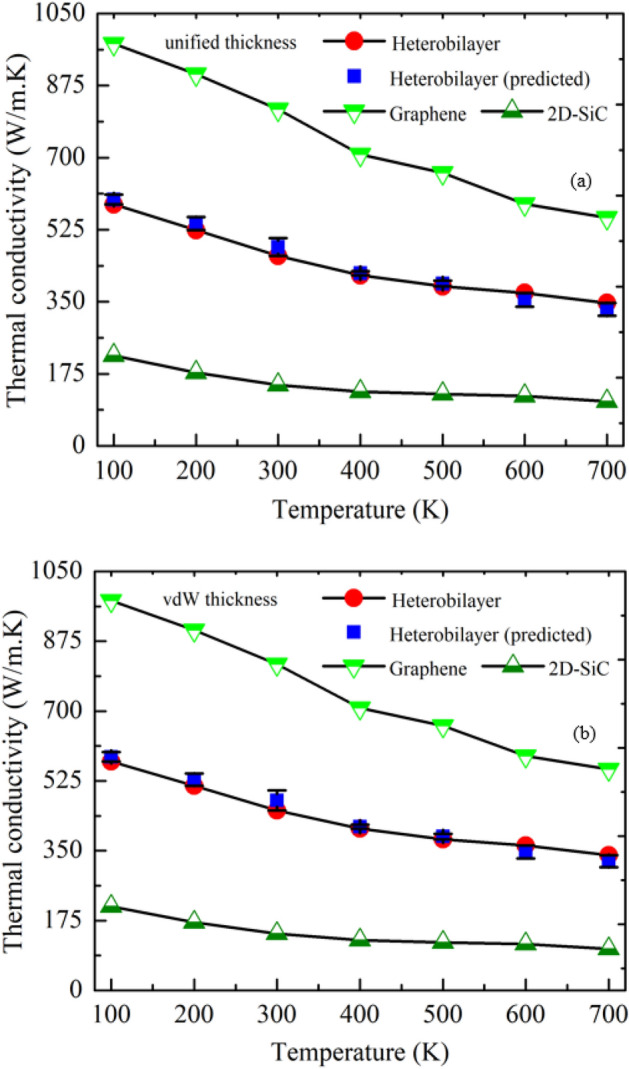
Temperature dependence of thermal conductivity of graphene, 2D-SiC and the hetero-bilayer from 100 to 700 K at (**a**) unified thickness and (**b**) vdW thickness (Coupling strength χ = 1). Each data point is averaged from three independent simulations with different initial conditions.

To investigate the temperature effect on *κ*, a widely varying temperature, ranging from100 K to 700 K and *χ* = 1, is considered. Figure 2 demonstrates the temperature dependence of in-plane *κ* for the vdWH as well as the 2D sheet of graphene and SiC. The in-plane thermal transport behavior of vdWHs is largely affected by the system thickness. Although, the height of a 2D system is normally taken as the thickness of that specific monolayer^26,50,51^, the total thickness of a vdWH system should be estimated carefully since there is an interlayer gap between the two materials. During the investigation, the overall thickness of the vdWH was measured as the summation of two distinct material thicknesses. The thicknesses of 2D-SiC and graphene were assumed to be *d*_*2D-SiC*_ = 3.5 Å, and *d*_*G*_ = 3.35 Å^34,52^. Hence, the total thickness of the graphene/2D-SiC vdWHs was 6.85 Å. Nonetheless, Wu et al.^53^ proposed that the sheet thickness of different 2D configurations does not change with alteration of the material. They discovered that the thickness of the graphene (3.35 Å) could be applied for computing thermal conductivity in all types of 2D structures. Consequently, both *d*_*G*_ and *d*_*2D-SiC*_ are considered to be 3.35 Å. In this perspective, the total thickness of the vdWH is 6.7 Å. We counted both of these thicknesses in our study, which we designated as vdW thickness (6.85 Å) and unified thickness (6.7 Å).

It is obvious from Fig. 2 that the in-plane *κ* is almost the same for both thicknesses. With increasing temperature, a monotonic decreasing trend of *κ* is observed for both vdWH and individual layer of graphene and 2D-SiC, as anticipated for phonon mediated heat conduction in nanomaterials. The maximum value of *κ* reaches to 586.47 W/mK at 100 K for the graphene/2D-SiC vdWH, and reduces to 346.81 W/mK at 700 K. The maximum drop of κ for graphene/2D-SiC vdWH, and individual sheet of graphene and 2D-SiC are found as 41.0%, 43.3%, and 39.8%, respectively. Several forgoing studies on 2D materials based vdWH also yielded similar results^5,54,55^. Ahammed et al.^5^ revealed that as the temperature rises from 100 to 700 K, the *κ* of stanene/2D-SiC heterostructure reduces from ~ 70 W/mK to ~ 24.81 W/mK. With increasing temperature from 100 to 600 K, the *κ* declines from ~ 365 W/mK to 150 W/mK for the stanene/Graphene heterostructure^41^,  ~ 1964.71 W/mK to ~ 438.19 W/mK for the graphene/$C_{3} N$ heterostructure^43^, and ~ 9 W/mK to ~ 2 W/mK for the stanene/silicene hetero-bilayer^56^, which all are in well agreed with our findings. Moreover, similar outcomes were also reported for heterostructures based on MoS_2_ and MoSe_2_^57,58^.

**Figure 3:**
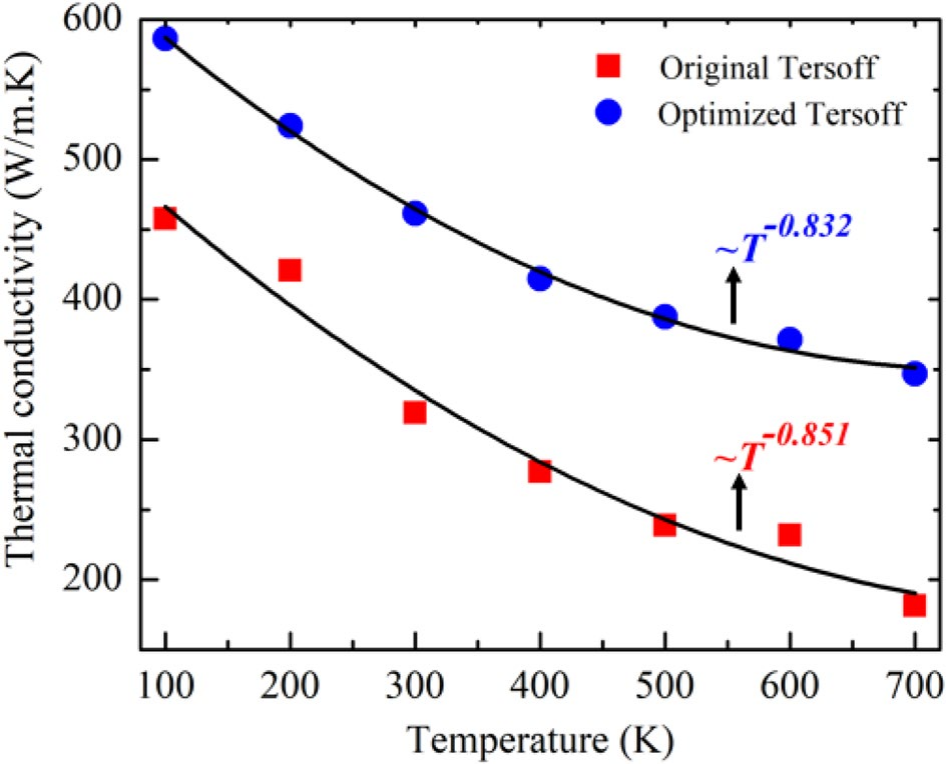
Temperature dependence of the thermal conductivity of the graphene/2D-SiC hetero-bilayer for two different potentials.

Though the estimation of *κ* of heterobilayer is based on the Fourier law, we observe that the *κ* digresses from the standard 1/*T* law at high temperatures. As interatomic force fields have a crucial effect on *κ*, we calculate the temperature dependent *κ* behavior for two different potentials. As demonstrated in Fig. 3, *κ* differs slightly from the standard 1/*T* law for both types of potentials. The amount of divergence from the Fourier law was also calculated, yielding $T^{ - 0.832}$ and $T^{ - 0.851}$ for the optimized and initial Tersoff potentials, respectively. This type of slow reduction rate of *κ* at high temperatures has also been observed in Si/Ge superlattice^59^, stanene/2D-SiC^5^ vdWH, single layer 2D-SiC sheet^34^ and monolayer GaN^35^. As mentioned in Ref.^60^, the enhanced phonon–phonon Umklapp scattering causes this 1/T^α^ trend. Moreover, Qin et al.^35^ demonstrated that the contribution of high-frequency phonons may increase with increasing temperature, leading to this slow rate reduction behavior.

To understand this unusual behavior, the temperature dependent phonon spectrum was calculated. The phonon density of states (PDOS) was measured by means of Fourier transform of velocity auto-correlation function (VACF)^6^. We calculated the VACF according to

5 $$ Z_{\alpha } \left( t \right) = \frac{{\left\langle {V_{i\alpha } \left( 0 \right) \cdot V_{i\alpha } \left( t \right)} \right\rangle }}{{\left\langle {V_{i\alpha } \left( 0 \right) \cdot V_{i\alpha } \left( 0 \right)} \right\rangle }} $$

where $V_{i\alpha } \left( 0 \right)$ and $V_{i\alpha } \left( t \right)$ are the *i*^th^ particle velocities of element α (C or Si) at time 0and *t*, respectively. The PDOS was determined from the VACF according to following formula:

6 $$ F\left( \omega \right) = \frac{1 }{{\sqrt {2\pi } }}\mathop \smallint \limits_{ - \infty }^{\infty } dte^{i\omega t} Z_{\alpha } \left( t \right) $$

where *F*(ω) stands for the phonon spectrum at the vibrational frequency of ω.

**Figure 4:**
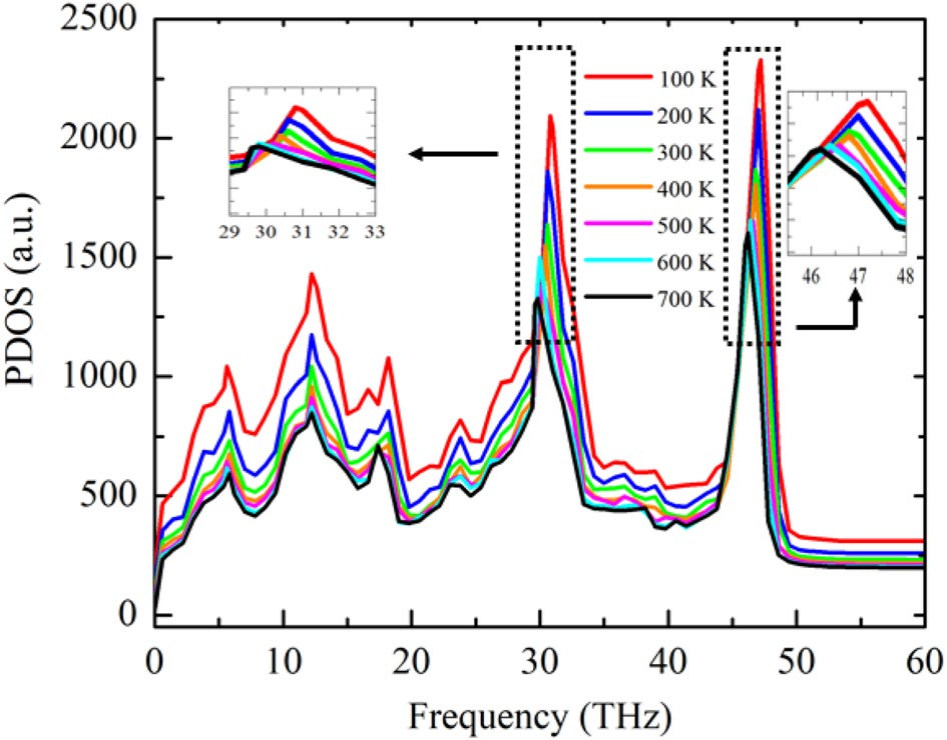
Effect of temperature on the in-plane phonon density of states (PDOS).

Figure 4 depicts the temperature-dependent in-plane PDOS for the graphene/2D-SiC vdWH. The vast portion of in-plane phonons inhabit the high-frequency areas, while a part occupies the low-frequency areas, as shown in Fig. 4, and all of these play a role in the heat energy transmission in this nanomaterial. As the figure demonstrates, the intensity of PDOS peaks reduces with increasing temperature. The reduction of peak intensities complies the decay relation of the *κ* with temperature. Moreover, a redshift is perceived from the fourth peak (29-31THz) and fifth peak (46-48THz) of the PDOS curve. This redshift indicates that the phonon energy decreases at increasing temperatures, which might cause the reduction in *κ* for graphene/2D-SiC vdWH. In 2D atomic crystals, low-frequency acoustic phonons play a substantial role in the transmission of heat energy. However, at high temperatures, the contribution of low-frequency acoustic phonons are reduced^5^. High-frequency optical phonons predominate at elevated temperatures, resulting in a generation of Umklapp phonon scattering^5,34,35^. Generally, the Umklapp phonon scattering enhances as the temperature of a crystal increases. This leads to the shortening of the mean free path (MFP) of phonons, resulting in a limited phonon heat conduction^43^.

**Figure 5:**
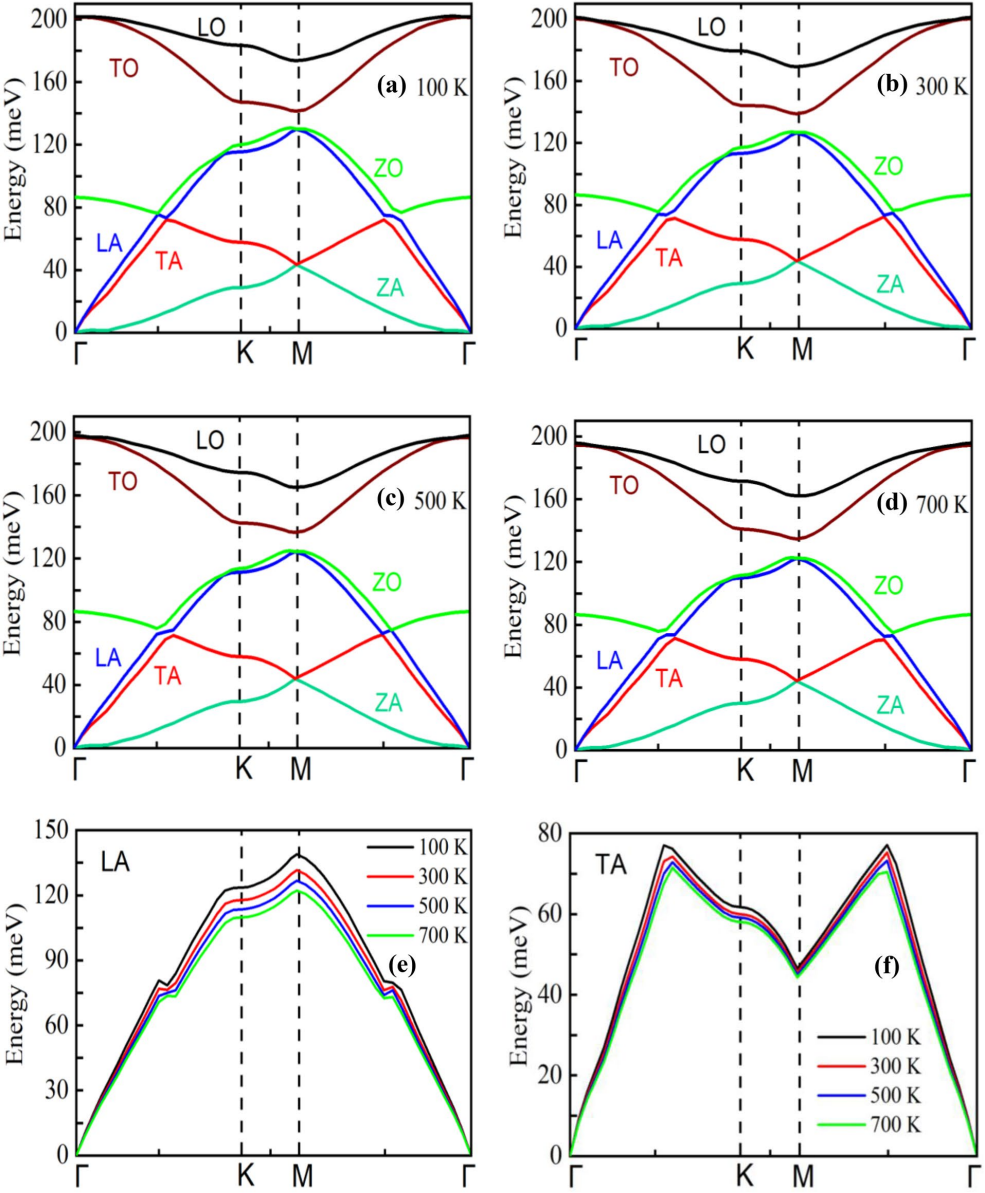
Phonon-dispersion relation for graphene/2D-SiC vdWH at (**a**) 100 K, (**b**) 300 K, (**c**) 500 K, and (**d**) 700 K. Temperature dependence of (**e**) LA and (**f**) TA mode phonons.

**Figure 6:**
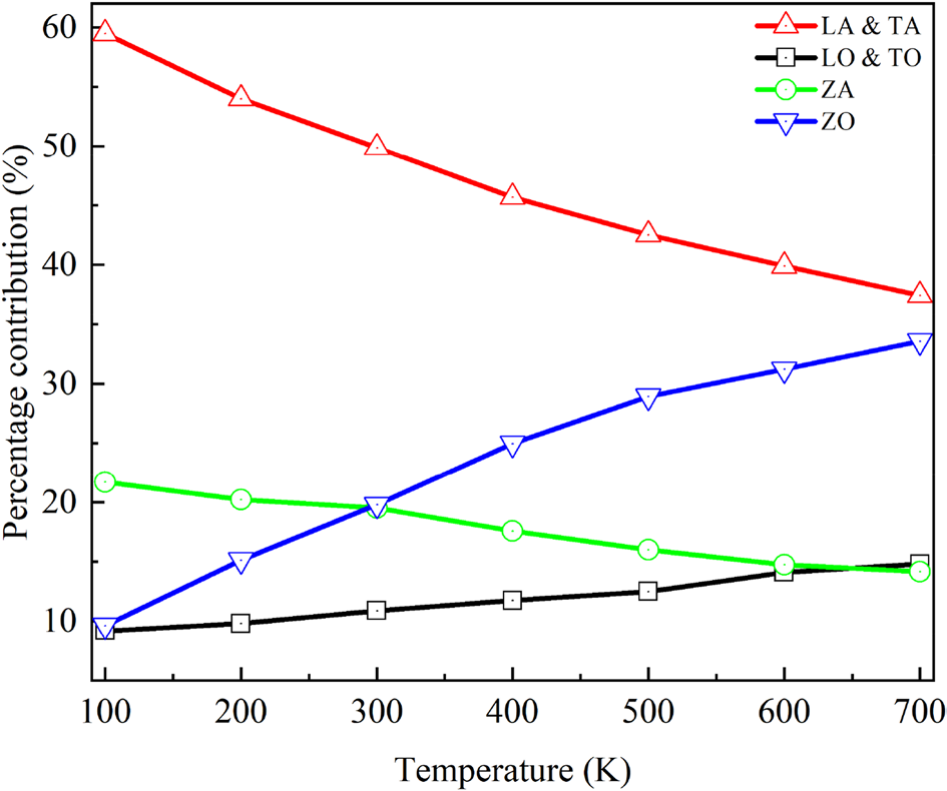
The percentage contribution of various phonon modes as a function of temperature.

To further explain the temperature effects on *κ,* we calculated the temperature dependent phonon-dispersion relation, as depicted in Fig. 5. The phonon-dispersion relation is calculated using the Fix phonon command of LAMMPS package developed by Kong et al.^61^ As shown in Fig. 5a–f, the low-frequency phonon mode, particularly the LA phonon mode, shows a softening nature with increasing temperature, causing the lower κ. High-frequency phonon modes such as LO and TO modes also show a downward shifting trend with increasing temperature, which may contribute to a reduction in *κ*. We also estimated the percentage contribution of each phonon mode with increasing temperature, as shown in Fig. 6. The contribution of LA and TA modes phonon decreases with increasing temperature. However, the percentage contribution of high frequency mode, especially the ZO phonon, increases significantly. Besides, a slight increase of LO and TO mode phonons is also observed. The enhanced contribution of high-frequency optical phonons at high temperatures may cause this slow rate reduction of *κ*. There could be other reasons for this minor deviation, because such a slight deviation is fairly common in defective structures (here heterostructure), as described in Refs^62,63^.

**Figure 7:**
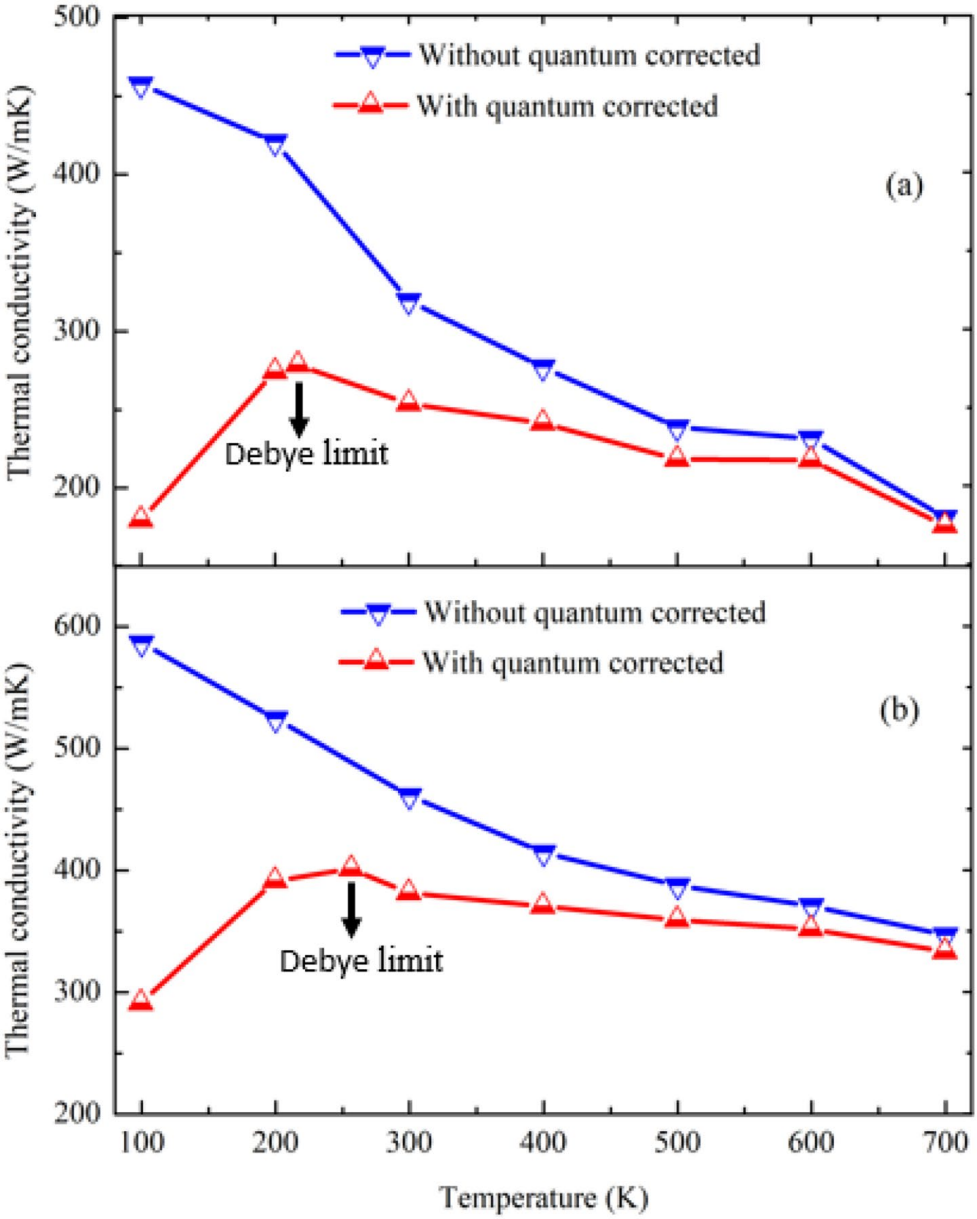
Quantum corrected thermal conductivity of the graphene/2D-SiC hetero-structure [(**a**) original (**b**)-optimized Tersoff].

At low temperatures, atomic vibrations remain in a resting place, also known as a frozen state, and remain in that state until the surrounding temperature is raised to a proper level. The sufficient temperature level is known as the Debye temperature level, and the *κ* estimated from the MD simulation up to this point is not fully accurate. Ultimately, to study low-temperature *κ*, quantum corrections are required. Using the PDOS of the graphene/2D-SiC heterobilayer, we first calculated the normal mode-specific heat. At constant volume, the specific heat ($$C_{V} )$$ can be defined as^34^ $$C_{V} = \frac{{3N{\rm K}_{B} \int\limits_{0}^{\infty } {\frac{{u^{2} e^{u} }}{{\left( {e^{u} - 1} \right)^{2} }}} F\left( \omega \right)d\omega }}{{\int\limits_{0}^{\infty } {F\left( \omega \right)d\omega } }}$$, where *F*(ω) signifies the phonon spectrum, $${\rm K}_{B}$$ is the Boltzmann constant, and *u* = $$\frac{{\hbar {\upomega }}}{{{\rm K}_{{\text{B}}} T}}$$. The simulated specific heat for the graphene/2D-SiC vdWH is depicted in Fig. S3 (supplementary information). It is obvious that as the temperature rises, phonon modes are excited, leading to increased specific heat behavior. The $$C_{V}$$ reaches the steady state due to the complete stimulation of low-frequency phonons at high temperatures. Incorporating the quantum correction at low temperatures *κ* can be estimated according to $$\kappa_{qc} = \frac{{C_{v} }}{{3NK_{B} }} \times k_{MD}$$, where $$k_{MD}$$ is the MD simulated thermal conductivity and $$\kappa_{qc}$$ is the thermal conductivity after quantum correction. The calculated $$\kappa_{qc}$$ for the optimized and original Tersoff potentials are presented in Fig. 7. For both potentials, the $$\kappa_{qc}$$ follows a growing trend up to the Debye limit, and then a progressively reducing trend as temperature rises. The calculated Debye limit were found as 257 and 217 K for the optimized and original Tersoff potentials, respectively. The rise in $$\kappa_{qc}$$ is owing to the rising nature of the specific heat, coming from the increased quantity of excited phonons up to the Debye limit. Again, up to the Debye limit, the phonon group velocities are assumed to be constant. On the other hand, the saturated nature of $$C_{V}$$ has a minimal effect on $$\kappa_{qc}$$ at high temperatures, especially after crossing the Debye threshold. Hence, phonon–phonon scattering, particularly Umklapp scattering at high temperatures, is considered to have a strong *κ*-lowering effect. As a result, beyond the Debye limit, the quantum corrected results show a declining trend in thermal conductivity. Furthermore, when compared to the $$k_{MD}$$, the $$\kappa_{qc}$$ exposes a relatively limited value up to the Debye limit due to the reduced quantity of excited phonons.

Although we calculated the *κ* considering the linear region of the temperature profile as described in most previous NEMD simulations, a recent study^64^ demonstrated that the nonlinear portion should also not be ignored when calculating thermal transport of finite system. Here, we also calculated the *κ* by considering the average temperature of the thermostats to incorporate the nonlinear region of the temperature profile. The calculated temperature dependent *κ* while considering the average temperature of heat source and sinks is depicted in Fig. S4 (supplementary information). The maximum value of *κ* reaches to 523.93 W/mK, 885.43 W/mK, and 178.65 W/mK at 100 K for the graphene/2D-SiC vdWH, graphene, and 2D-SiC, respectively, and reduces to 259.10 W/mK, 421.10 W/mK, and 77.10 W/mK at 700 K. It is apparent that at all temperatures, the *κ* is slightly smaller than the estimated *κ* using only the linear portion, which is in line with the demonstration in Ref^64^. This result also implies that the temperature drop close to the thermostat is physical in finite system, as stated in Refs^65,66^.

**Figure 8:**
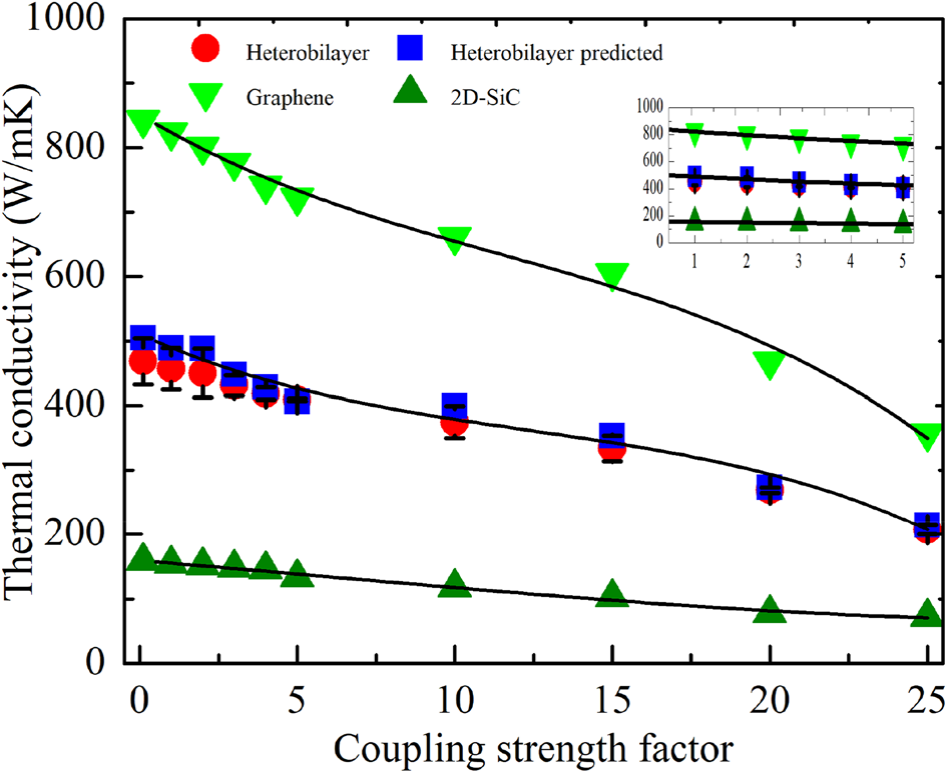
Dependence of in-plane thermal conductivity with interlayer coupling strength *χ*.

Improving thermal transport is crucial for novel materials to be used in nanodevices. Various approaches, such as modulating the interface interaction or varying the rotational angle of substrate, have recently been discovered to improve the thermal transport behaviors of vdWHs^40–43,49,57,67^. The interlayer coupling has long been known to have a substantial impact on the thermal transport characteristics. The LJ coupling strength χ was used to study the interfacial contact pressure dependent *κ* for graphene/2D-SiC vdWH. A wide variety of χ values ranging from 0.1 to 25 were chosen to determine the coupling strength dependence of *κ.* First, the stability of the structures was checked for these diverse values of χ and then the *κ* dependence of χ was calculated. Figure 8 represents the variation of the calculated thermal conductivities against χ. Three separate simulations with varied starting conditions were performed and averaged to extract each data point. As χ increases from 0.1 to 25, the *κ* of graphene/2D-SiC decreases monotonically from 468.62 to 207.09 W/mK. The *κ*, for the independent layer of graphene, drops dramatically from 842.88 to 357.23 W/mK, while the *κ* for 2D-SiC drops steadily from 158.32 to 71.82 W/mK. The flexural mode phonons of 2D-SiC constrain the flexural mode phonons of graphene, resulting in a reduction in the *κ* of the graphene/2D-SiC vdWH when compared to the independent layer of graphene or 2D-SiC sheet. With increasing χ, restrictions between the flexural mode phonons of the two layers increases as the interaction of the graphene layer and 2D-SiC layer is enhanced^40–43,67^. Accordingly, the *κ* of graphene, 2D-SiC, as well as of the graphene/2D-SiC vdWH decreases as the restriction is increased.

**Figure 9:**
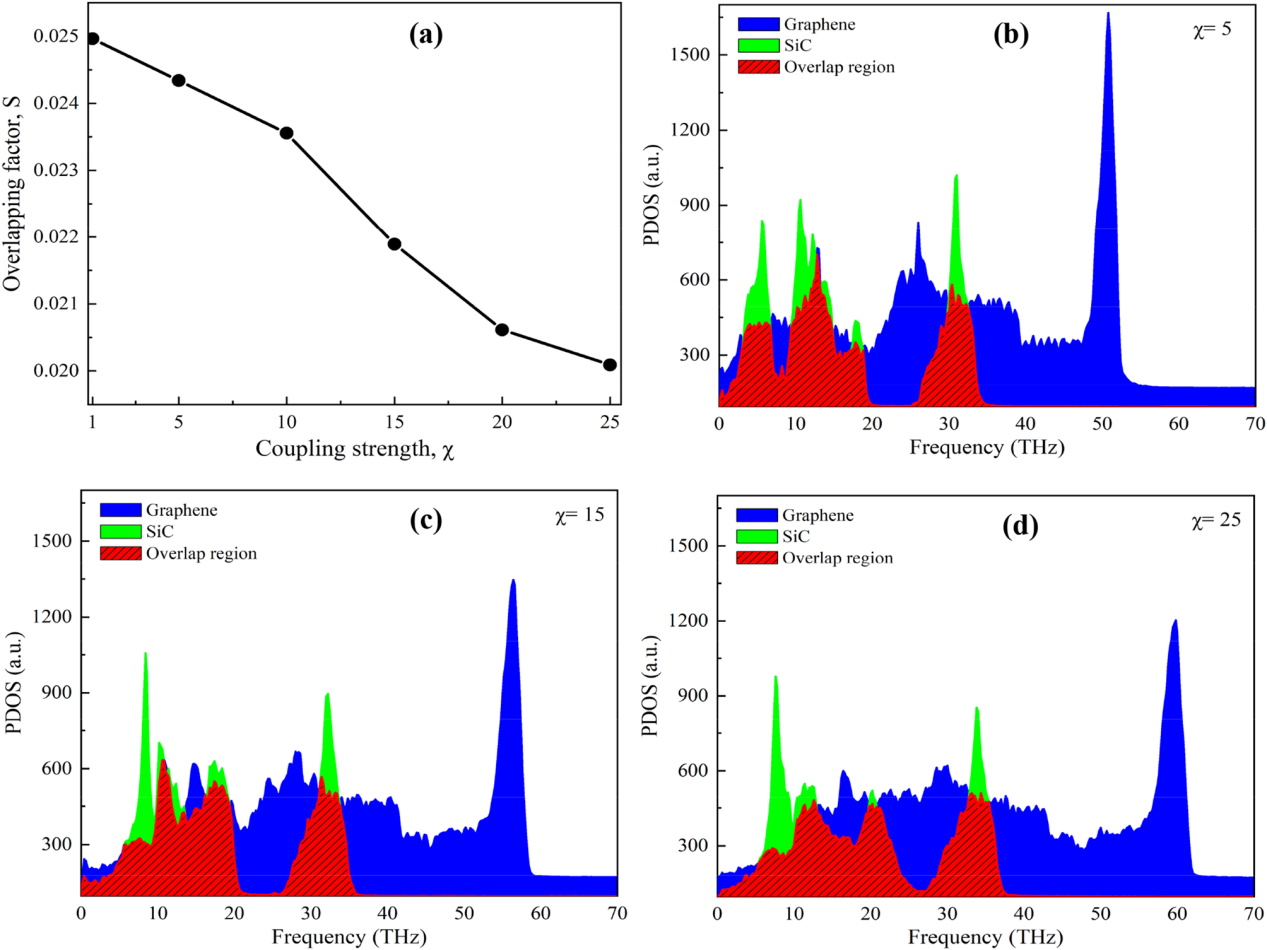
(**a**) Calculated overlap factor *S* as a function of interlayer coupling strength *χ*. (**b**)–(**d**) Overlapping region of PDOS between graphene and 2D-SiC as a function of *χ*. The slant areas (red) denote the overlap region between graphene and 2D-SiC PDOS.

Moreover, to have a better understanding of the χ dependence of *κ*, we calculated the phonon overlapping factor (*S*) of graphene/2D-SiC heterobilayer, which demonstrates the capacity of thermal energy exchanging between graphene and 2D-SiC phonon. The following equation is used to compute the overlapping factor *S*:

7 $$ S = \frac{{\mathop \smallint \nolimits_{ - \infty }^{\infty } F_{graphene} \left( \omega \right) \cdot F_{2D - SiC} \left( \omega \right)d\omega }}{{\mathop \smallint \nolimits_{ - \infty }^{\infty } F_{graphene} \left( \omega \right)d\omega \cdot \mathop \smallint \nolimits_{ - \infty }^{\infty } F_{2D - SiC} \cdot d\omega }} $$

where, the subscripts "graphene" and "2D-SiC" denote all the phonon modes of graphene and 2D-SiC, respectively. The *S* shows a decreasing trend ranging from ~ 0.026 to ~ 0.020 with the increase of χ from 0.1 to 25, as depicted in Fig. 9a–d.The decrease in overlapping area reflects a decrease in the capacity to exchange thermal energy between graphene and 2D-SiC^68,69^. Due to the increase of phonon scattering between two layers with increasing χ, the capacity of exchange thermal energy reduces. It can also be observed that the PDOS of graphene is slightly flattened with increasing χ. Such flattening can broaden some phonon modes, potentially reducing phonon lifetime and MFP^70^. Eventually, this may reduce the overall thermal conductivity of the heterobilayer.

**Figure 10:**
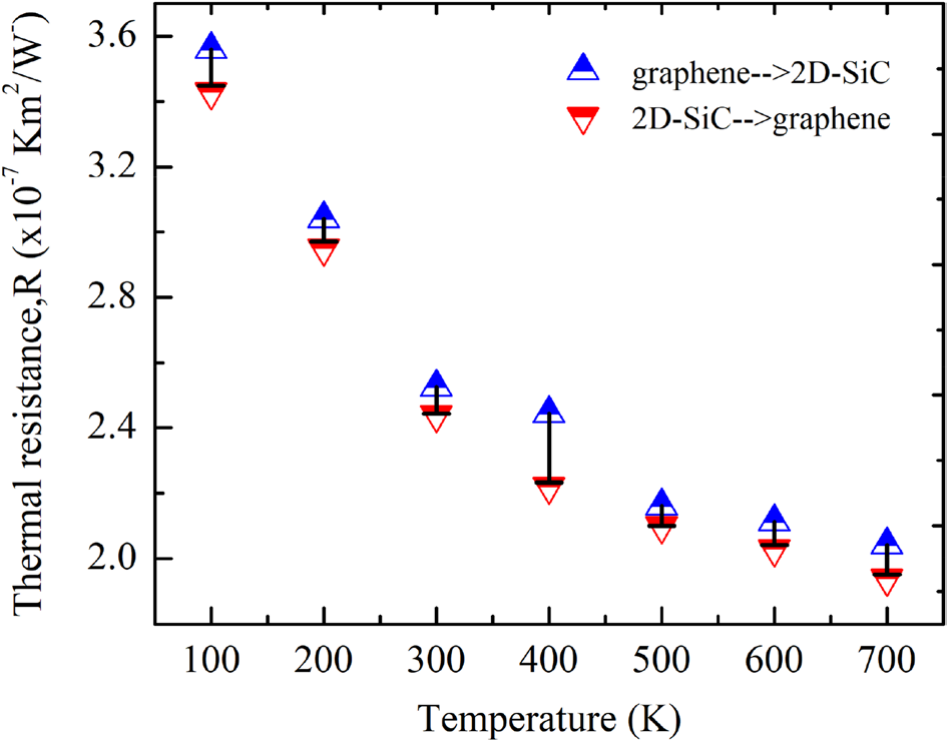
Interfacial thermal resistance variations in the heterobilayer for different temperatures from 100 to 700 K. Here, GRA- > SiC and SiC- > GRA refer to the direction of heat flow from graphene to SiC and SiC to graphene, respectively. There is a slight thermal rectification observed in the out-of-plane thermal conduction for different temperatures.

**Figure 11:**
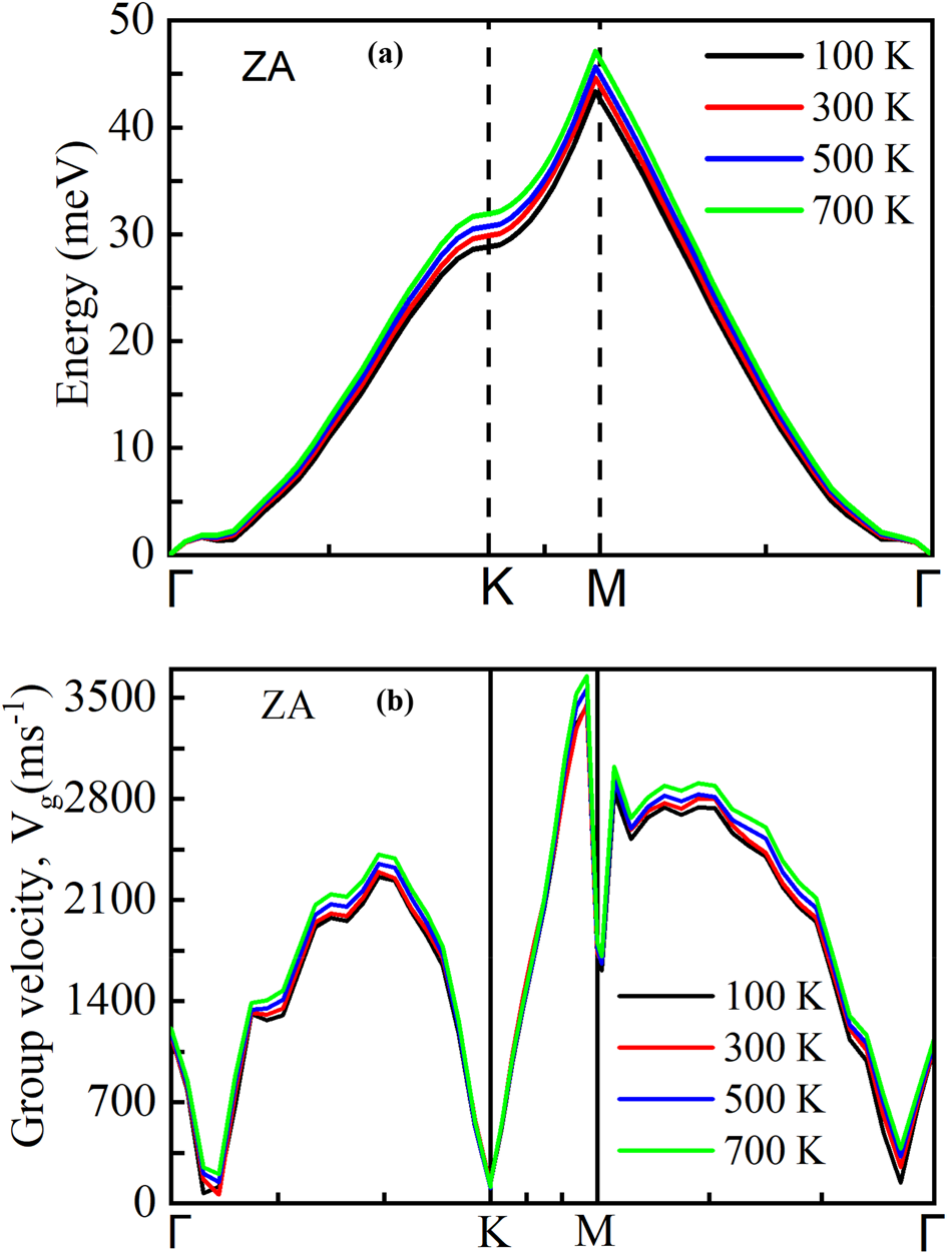
(**a**) Variation of the phonon-dispersion relation and (**b**) phonon group velocity of the ZA mode phonon at different temperatures.

Although 2D materials have superior in-plane heat transport behavior, out-of-plane heat transport is often the barrier for thermal energy exchange due to the poor vdW interaction at the interface. The ability to control the heat flow has led to possible fascinating applications, which strongly depends on the better understanding of the thermal conduction mechanism at the interface. The temperature dependence of *R* at χ = 1 is depicted in Fig. 10. The estimated *R* reaches to a maximum value ~ 3.559 $\times 10^{ - 7} Km^{2} W^{ - 1}$ at 100 K and monotonically reduces to ~ 2.041 $\times 10^{ - 7} Km^{2} W^{ - 1}$ at 700 K. Earlier investigations showed that inelastic scattering affects the most of the energy transport at vdW interface compared to the elastic scattering, at high temperatures^57^.This is because high-frequency phonons may disintegrate into multiple low-frequency phonons at high temperatures, causing the enhancement of inelastic scattering probability. Low-frequency phonons have a better chance of being transmitted through the interface than high-frequency phonons. As a result, phonon transmission coefficients at the interface are enhanced, and *R* is decreased. Anharmonicity within the materials is discovered to have an essential role in heat transportation at interface by enhancing energy transmission between distinct phonon modes. Wu *et* al.^71^ explored the influence of anharmonicity in heat transfer across an interface composed of monatomic and diatomic lattices. At higher temperatures, increased anharmonic scattering results in more effective energy redeployment to low-frequency phonons, which can more efficiently transmit heat across the interface, resulting in a reduced *R*. To further explain the temperature effects on *R*, we calculated the temperature dependent out-of-plane phonon-dispersion and phonon-group velocities. As depicted in Fig. 11a, the intensity of ZA mode phonon shows an increasing nature with increasing temperature. It indicates that as the temperature rises, more phonons move from one layer to the next, increasing thermal conductivity in the out-of-plane direction and decreasing *R*. The estimated group velocity for ZA mode phonon as shown in Fig. 11b, also shows an increasing nature with increasing temperature, further verifying the lowering behavior of *R* as temperature rises.

**Figure 12:**
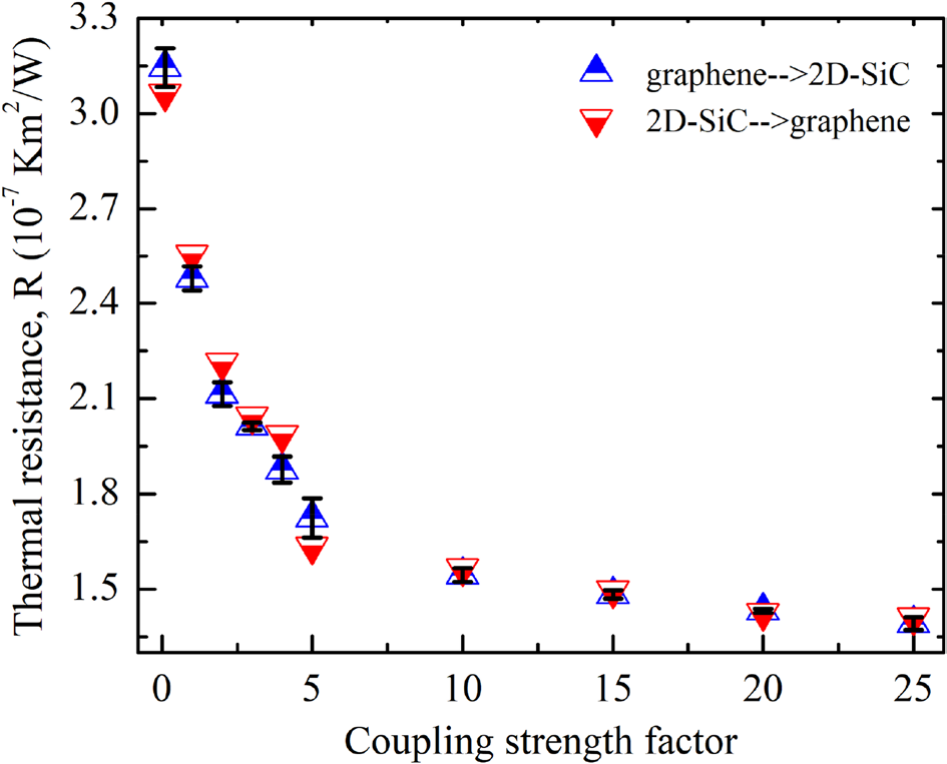
Interfacial thermal resistance variations with different coupling strength factors from 0.1 to 25. There is no thermal rectification observed in the out-of-plane thermal conduction for different coupling strengths.

**Figure 13:**
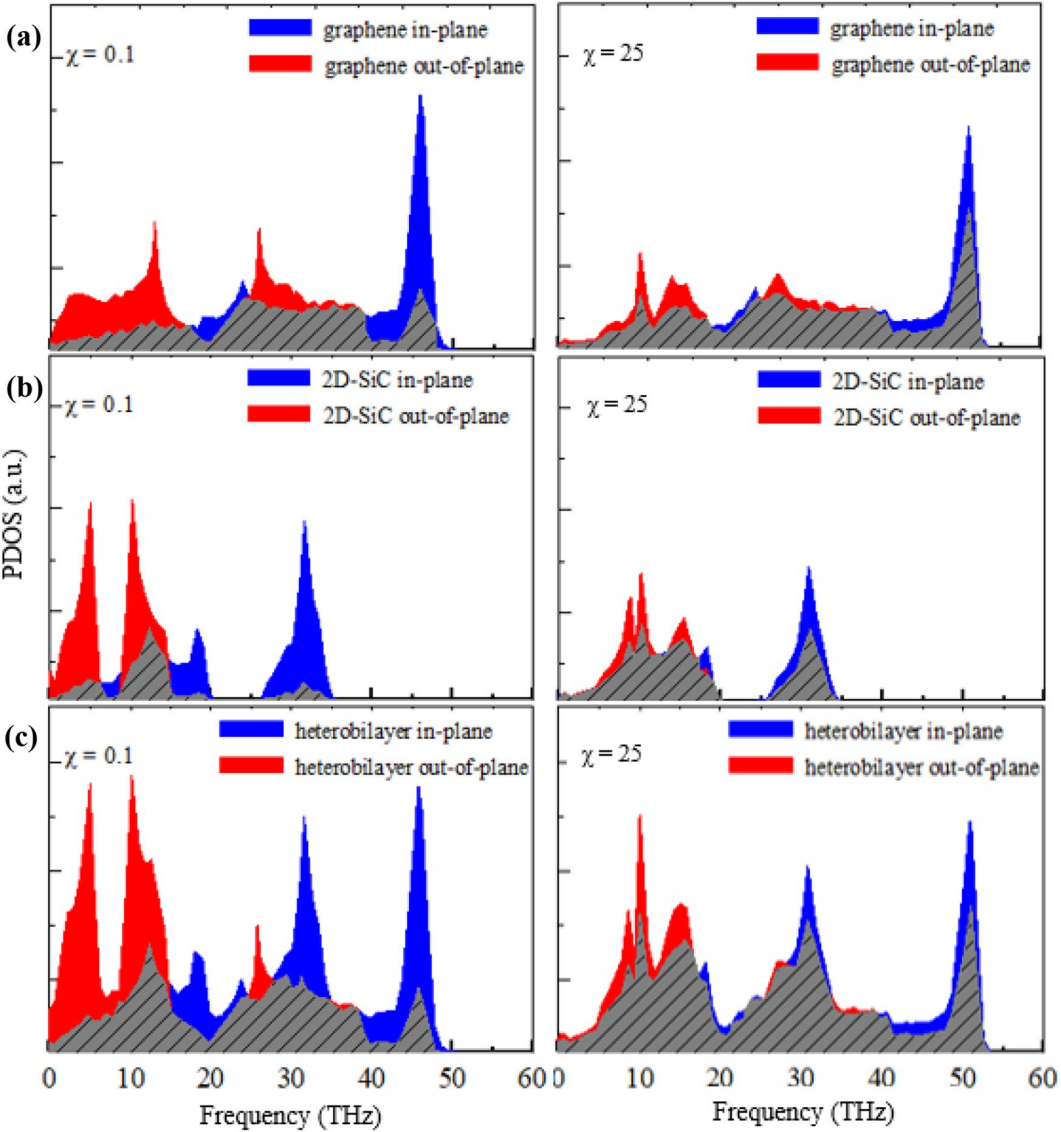
PDOS of (**a**) graphene, (**b**) 2D-SiC, and (**c**) the graphene/2D-SiC heterobilayer in in-plane and out-of-plane directions with χ = 0.1 and χ = 25. The slanted line areas denote the overlap of the in-plane and out-of-plane PDOS.

Next, we study the χ dependence of *R* on the graphene/2D-SiC vdWH. Figure 12 illustrates the influence of χ on *R* at 300 K. The computed *R* drops monotonically when χ varies from 0.1 to 25. Usually, boosting the χ increases the interaction at the interface, which enhances the coupling of phonons between SiC and graphene layers and unswervingly raises transmission of phonon probability across the contact. Moreover, atoms in one surface might be scattered more strongly by the other layer’s atoms with increasing χ, leading to the increasing of interface scattering. The capability of energy interchange between in-plane and out-of-plane phonons is manifested by calculating the overlap in the PDOS as shown in Fig. 13. With χ = 0.1, the computed *S* for graphene, 2D-SiC, and the graphene/2D-SiC vdWH is 0.022, 0.026, and 0.023, respectively, and increases to 0.026, 0.034, and 0.028 when χ = 25. It indicates that the interfacial thermal transfer capacity grows when the coupling of in-plane and out-of-plane phonons improves. Because of the scattering of high frequency phonons, the *S* of graphene has a slight improvement. The *R* of the system decreases as a result of the improved coupling strength. Therefore, we conclude that interlayer coupling strength may efficiently tune the thermal resistance across the graphene/2D-SiC vdW interface.

## Conclusions

In conclusion, we used NEMD and TPP simulations to systematically investigate temperature and contact pressure induced thermal transport across graphene/2D-SiC vdWH. The computed in-plane *κ* was found to be considerably reduced by system temperature, with maximum reductions of43.3%, 39.8%, and 41.0% for graphene, 2D-SiC, and the bilayer, respectively. Different from the typical *1/T* law, an abnormal *κ* for the graphene/2D-SiC bilayer was observed at high temperatures. A coupling strength dependent tunable *κ* was observed for this heterostructure, which is advantageous for nanodevice production since thermal performance may be adjusted by contact pressure. The calculated *R* was also found to decrease monotonically with increase of the temperature and coupling strength. The heat conduction mechanism across the heterostructure interface was demonstrated using phonon calculations. The cross-plane phonon interaction between the graphene and 2D-SiC layers is revealed to be the primary thermal channel for interfacial heat conduction. The Umklapp scattering converts the high-frequency phonons into more low-frequency phonons, which can subsequently be linked with the phonons in other layer and contributing to interface thermal transport and lowering *R*. Moreover, enhanced phonon coupling with increasing χ facilitates the heat transfer from graphene to SiC and thus bring about the decrease in *R*. This study reveals the mechanism of heat dissipation and interfacial engineering for thermal conductance manipulation in the graphene/2D-SiC vdW heterostructure.

## Supplementary Information

Supplementary Information.

## Data Availability

The data that support the findings of this study are available from the corresponding author upon reasonable request.
