# Supplementary material for: Temperature and interlayer coupling induced thermal transport across graphene/2D-SiC van der Waals heterostructure
Source: Sci Rep. 2022 Jan 14;12:761. doi: 10.1038/s41598-021-04740-4 (PMC8760313; doi:10.1038/s41598-021-04740-4)
Supplement: Supplementary file 1 — Supplementary Information. [file 41598_2021_4740_MOESM1_ESM.docx]

**Supplementary Information**

**Temperature and interlayer coupling induced thermal transport across graphene/2D-SiC van der Waals heterostructure**

Md. Sherajul Islam*^,a, c^, Imon Mia^a^, A. S. M. Jannatul Islam ^a^, Catherine Stampfl^b^, Jeongwon Park^c,d^

^a^Department of Electrical and Electronic Engineering, Khulna University of Engineering &Technology, Khulna 9203, Bangladesh

^b^School of Physics, The University of Sydney, New South Wales 2006, Australia

^c^Department of Electrical and Biomedical Engineering, University of Nevada, Reno, NV 89557, USA

^d^School of Electrical Engineering and Computer Science, University of Ottawa, Ottawa, ON K1N 6N5, Canada

Figure S1


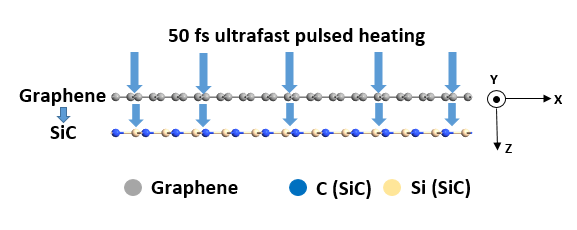


**d**

Figure S1. Schematic of the transient pump probe method to calculate the out-of-plane thermal conductivity. Here, d refers to the interlayer distance. A 50 fs ultra-fast heat impulse is applied to the graphene in the out-of-plane (*z*) direction to compute the interface thermal resistance.

Figure S2


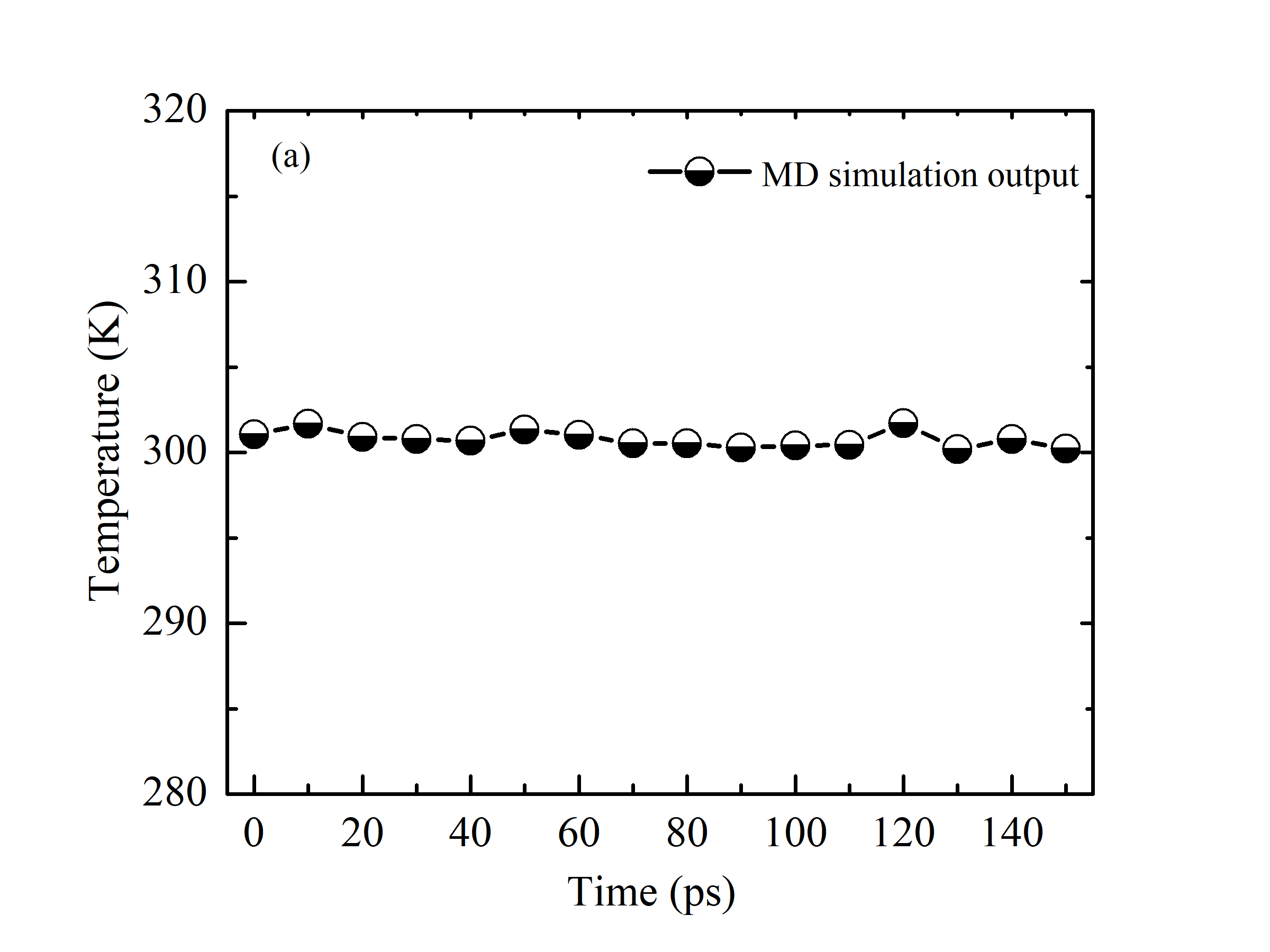


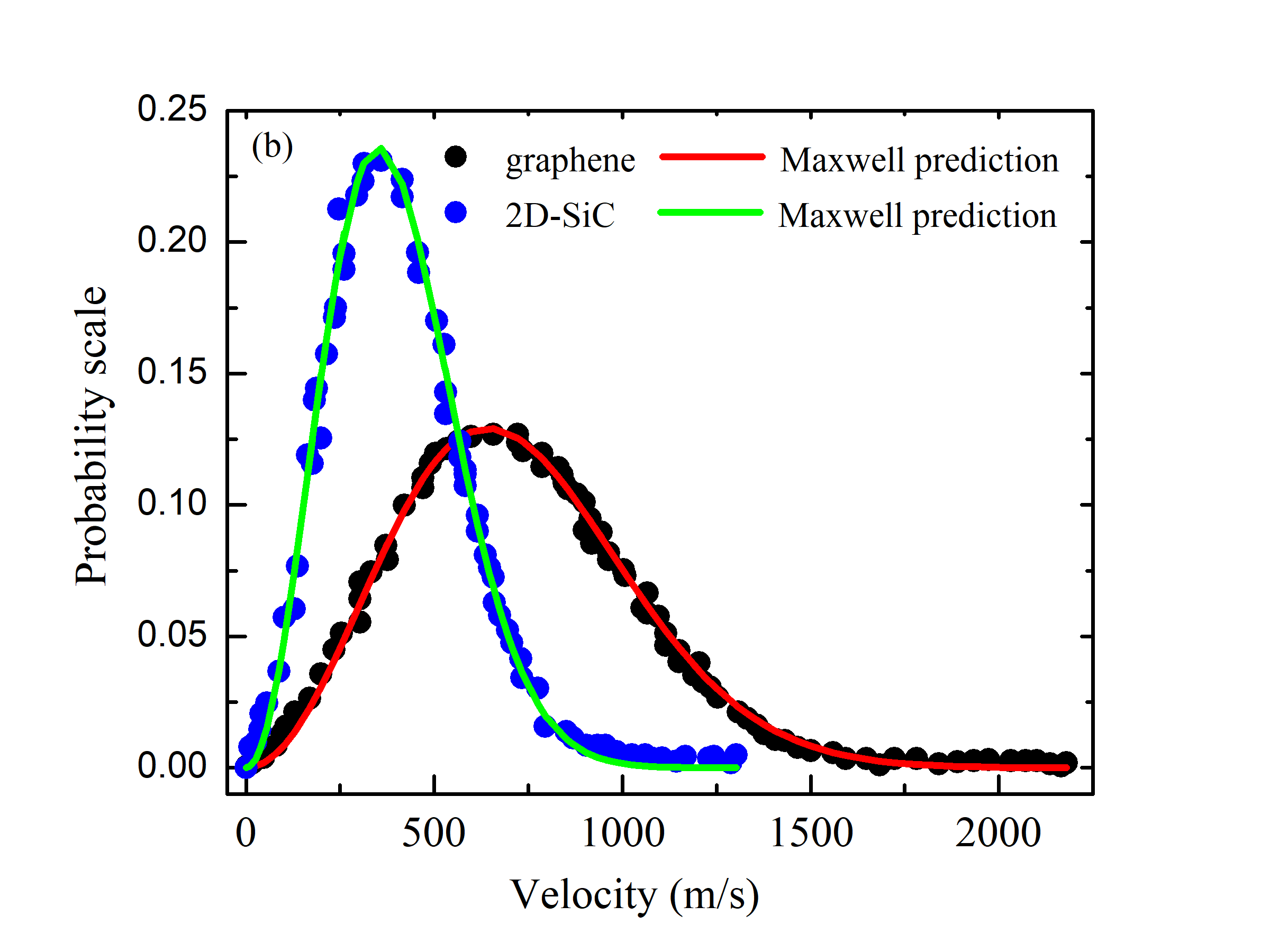


Figure S2. (a) Final relaxation profile of the system at desired temperature. (b) Comparison of the graphene and 2D-SiC atomic velocity distributions with the Maxwell theoretical prediction at steady state condition.

Figure S3


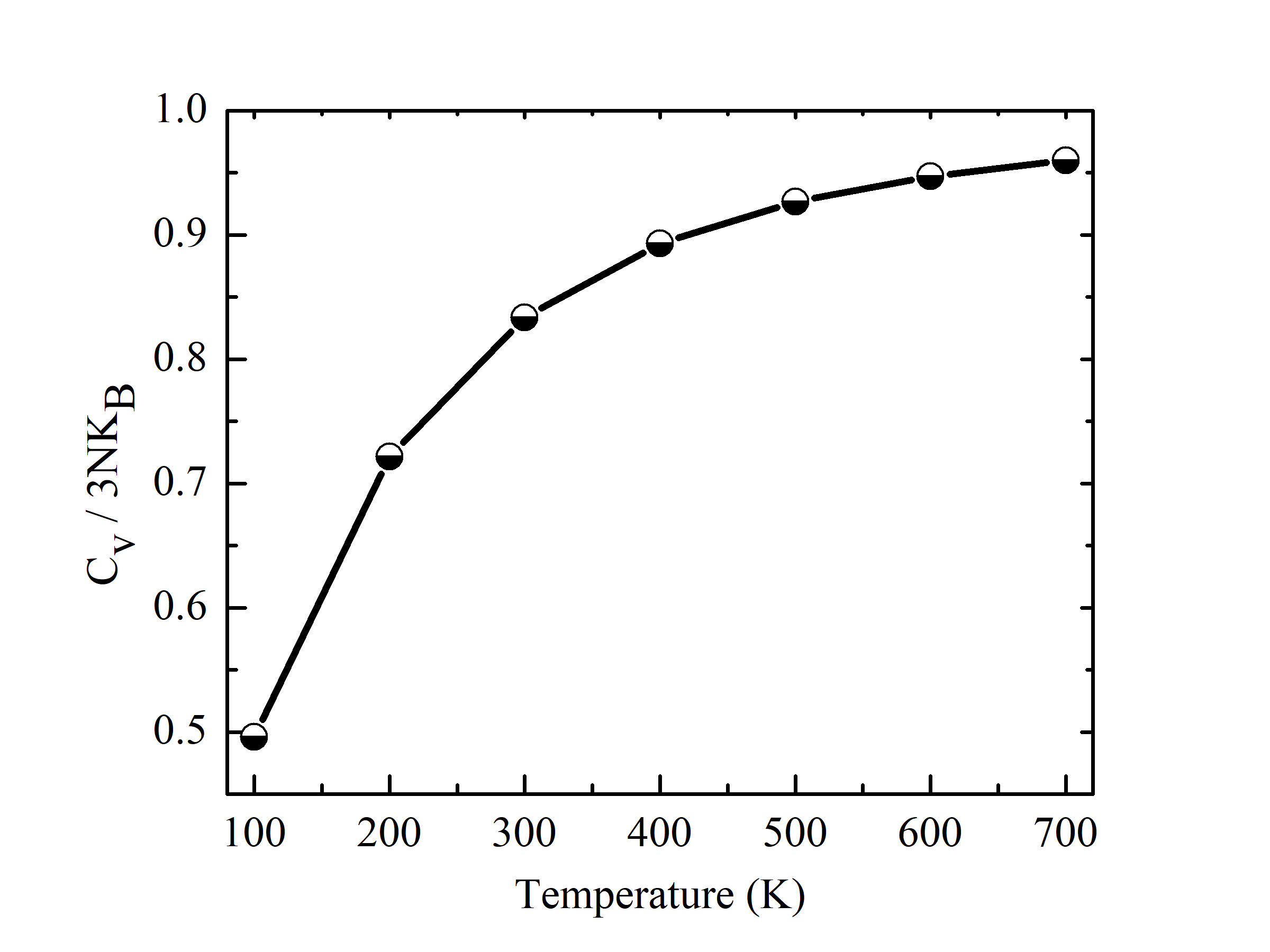


Figure S3. The calculated specific heat as a function of temperature for the graphene/2D-SiC vdWHs.

Figure S4

Figure S4. Temperature dependent thermal conductivity of graphene, 2D-SiC, and graphene/2D-SiC vdWHs for vdW thickness considering both linear and nonlinear parts of the temperature profile described in Fig. 1.
